# Differential Susceptibilities to BmNPV Infection of Two Cell Lines Derived from the Same Silkworm Ovarian Tissues

**DOI:** 10.1371/journal.pone.0105986

**Published:** 2014-09-15

**Authors:** Jun Zhang, Xue-Mei Chen, Chun-Dong Zhang, Qian He, Zhan-Qi Dong, Ming-Ya Cao, Xiao-Long Dong, Cai-Xia Pan, Cheng Lu, Min-Hui Pan

**Affiliations:** 1 State Key Laboratory of Silkworm Genome Biology, Southwest University, Chongqing, China; 2 Institute of Life Sciences, Chongqing Medical University, Chongqing, China; 3 Department of Biochemistry and Molecular Biology, Chongqing Medical University, Chongqing, China; University of Minnesota, United States of America

## Abstract

We previously established and characterized two insect cell lines (BmN-SWU1 and BmN-SWU2) from *Bombyx mori* ovaries. Here, we examined their differential susceptibilities to *Bombyx mori* nucleopolyhedrovirus (BmNPV) despite having originated from the same tissue source. BmN-SWU1 cells were susceptible and supported high titers of BmNPV replication, while BmN-SWU2 cells were resistant to BmNPV infection. Subcellular localization analysis demonstrated that very few BmNPV particles could be imported into BmN-SWU2 cells. However, initiation of BmNPV DNA replication but not amplification was detected in BmN-SWU2 cells after transfection with vA4^prm^-VP39-EGFP bacmid DNA. BmNPV transcription assays showed that late and very late but not early viral genes apparently were blocked in BmNSWU2 cells by unknown mechanisms. Further syncytium formation assays demonstrated that the BmNPV envelope fusion protein GP64 could not mediate BmN-SWU2 host cell-cell membrane fusion. Taken together, these results indicate that these two cell lines represent optimal tools for investigating host-virus interactions and insect antiviral mechanisms.

## Introduction

The domesticated silkworm, *Bombyx mori*, is the lepidopteran model species and is an economically important insect used for silk production and proteinaceous drug expression [1], [2], [3]. However, *Bombyx mori* nucleopolyhedrovirus (BmNPV), as a natural viral pathogen for the silkworm, causes heavy larval mortality and enormous losses to the sericulture industry. Furthermore, no effective therapeutic means are available currently to control BmNPV infection. BmNPV belongs to the *Baculoviridae* family of rod-shaped enveloped viruses. During the baculovirus infection cycle, it produces two types of virions, budded viruses (BVs) and occlusion-derived viruses (ODVs). These two virion types contain identical genomic information and nucleocapsid structures but different viral envelopes, as they are produced at different stages of the virus life cycle. BVs are responsible for systematic infection within the silkworm, while ODVs mediate horizontal transmission between hosts [4], [5].

Baculovirus BVs enter host cells via a receptor-dependent endocytosis pathway at approximately 1 h post-inoculation (p.i.) [6]. For the lepidopteran baculovirus, the major viral envelope protein GP64 mediates a low-pH-triggered membrane fusion event [7]. GP64 also is essential for viral attachment to host cells and budding of progeny BVs from the surface of infected cells [8]. The cellular receptor for baculovirus BV attachment has not yet been identified, although a prior study identified a GP64 subdomain which is necessary for baculovirus-host receptor binding [9]. Other studies demonstrated that the pre-transmembrane (PTM) domain, transmembrane (TM) domain and long cytoplasmic tail (CT) domain of the GP64 protein play critical roles in cellular receptor binding, membrane fusion, virus budding or infectivity [9], [10], [11]. In addition, three putative cholesterol recognition domains (CRAC) were identified in GP64, which are important for anchoring the virus at the mammalian cell membrane [12], [13]. Therefore, surface cholesterol was hypothesized to be involved also in baculovirus binding to host insect cells.

Upon entry into the cytosol of target cells, baculovirus nucleocapsids move intracellularly to their replication sites using actin-based mechanisms, which require the viral P78/83 capsid protein and the host Arp2/3 complex [14], [15], [16]. Subsequently, virus transcription and DNA replication occur in the nuclei of infected cells. Expression patterns of the baculovirus genes are divided into four phases: immediate early, delayed early, late and very late phases. Early phase genes are necessary for viral DNA replication and transcription of late genes. The AcMNPV DNA replication initiates 5 to 6 h p.i. and peaks at approximately 18 h p.i. [17]. Other baculoviruses show slower replication cycles than AcMNPV. Because host programmed suicide is an effective antiviral strategy for virus-infected cells to significantly block virus replication, the baculovirus must shutoff this antivirus defense and manipulate the cellular machinery for viral gene transcription and genome replication [18], [19], [20].

Insect cell lines provide a platform for investigating baculovirus biology and a high expression system for extrinsic proteins. We previously established two cell lines from *B. mori* larval ovarian tissues, designated BmN-SWU1 and BmN-SWU2 [21]. In the present study, we confirmed the tissue origin of these cell lines and compared their levels of susceptibilities to BmNPV infection. We also investigated the defective steps in the infectious cycle of the virus in BmNPV-resistant BmN-SWU2 cells. These cell lines not only are tools for investigating virus-host interaction, but also represent models for: (1) identification of the BmNPV receptor and (2) investigations of resistance mechanisms that can be manipulated to block BmNPV infection.

## Materials and Methods

### Silkworm cell lines and tissues

Two ovarian cell lines BmN-SWU1 and BmN-SWU2 were isolated from the ovarian tissue of 3-day-old fourth instar *B. mori* larvae of the 21-872nlw strain. Briefly, *B. mori* primary ovarian cell cultures were explanted into a 25 cm^2^ flask at 27°C with 2 ml TC-100 insect medium (USBiological, Swampscott, MA, USA) supplemented with 20% (vol/vol) heat-inactivated fetal bovine serum (FBS, GE Healthcare, Piscataway, NJ, USA). Subcultured cell lines were propagated at 27°C in TC-100 insect medium supplemented with 10% FBS. The BmE-SWU1 cell line was derived from *B. mori* embryonic tissue and cultured in Grace's insect culture medium (GIBCO, Langley, OK, USA) supplemented with 10% FBS. Fresh ovarian tissues were obtained from 3-day-old fourth instar *B. mori* larvae of the 21-872nlw strain.

### Preparation of vA4^prm^-VP39-EGFP BVs

The recombinant BmNPV vA4^prm^-VP39-EGFP construct containing an EGFP reporter gene fused at the C terminus of an inserted copy of the major viral capsid protein VP39 controlled by the silkworm *actinA4* promoter was generated according to the manufacturer's instructions of the Bac-to-Bac Baculovirus Expression System (Invitrogen, Carlsbad, CA, USA). To construct the donor plasmid pFFA-A4-VP39-EGFP, we first digested pFastBacHTb with *Bam*HI and *Sna*BI to remove the polyhedrin gene promoter of AcMNPV. The resulting adhesive end was treated with Klenow polymerase. Self-ligation of the blunt fragment resulted in the plasmid pFFA. An *Eco*RI and *Spe*I fragment containing the *actinA4* promoter was amplified using the pA4LSV40 expression vector [22] as a template and inserted into *Eco*RI-*Sal*I digested pFFA to produce pFFA-A4^prm^. The *vp39* gene without a stop codon was PCR amplified from the BmNPV bacmid DNA, cleaved and subcloned into the *Sal*I site of pFFA-A4^prm^ to generate the plasmid pFFA-A4^prm^-VP39. The EGFP cassette was digested from the pEGFP-N1 plasmid (Clontech, Mountain View, CA, USA,) with *Sal*I and *Xba*I and subcloned downstream of the *vp*39 gene of the plasmid pFFA-A4^prm^-VP39 to generate pFFA-A4-VP39-EGFP. Subsequently, the donor plasmids were transformed into BmDH10Bac competent cells. Transposition of inserts from donor plasmids to the BmNPV bacmid was confirmed by diagnostic PCR as described by the Invitrogen manual. Finally, 1 µg of vA4^prm^-VP39-EGFP bacmid DNA was transfected into BmN-SWU1 cells using X-tremeGENE HP (Roche, Basel, Switzerland). At 5 days post-transfection (p.t.), supernatants of recombinant BVs were selected, amplified and titered using a 50% tissue culture infectious dose (TCID_50_) assay.

### Semi-quantitative RT-PCR (RT-PCR)

Expression patterns of *Bm-vasa* and *Bm-Integrin* were analyzed by RT-PCR. The *Bm-rpl3* gene encoding a silkworm ribosomal protein was used for normalization. RT-PCR was performed with primers for *Bm-vasa* (forward 5′ AAATAGGGGAAAACGGGGA 3′ and reverse 5′ CATACGGTCAGCCTCATCCAG 3′); *Bm-integrin* (forward 5' CTGCTTTAGAATCCACACCC 3' and reverse 5' ACAGTGGCAAATACCGCA 3'), *Bm-rpl3* (forward 5' TCGTCATCGTGGTAAGGTCAA 3') and reverse (5' TTTGTATCCTTTGCCCTTGGT 3'). The PCR procedure was conducted as follows: 94°C for 4 min, followed by 30 cycles of 94°C for 40 s, 55°C for 45 s, 72°C for 45 s, and 72°C for 10 min. The resulting PCR products were detected in a 1% agarose gel.

### Virus susceptibility

Susceptibility of ovarian cell lines to BmNPV infection was tested by exposure to vA4^prm^-VP39-EGFP BVs for 1 h at a multiplicity of infection (MOI) of 2 or 20. Infected cells were washed three times with TC-100 insect medium. Thereafter, the cells were cultured with medium supplemented with 10% FBS. This time point was designated as 0 h p.i. At various times after BmNPV infection, viral infection rates were determined by counting fluorescent signals in five different fields of each of the two cell lines under a fluorescence microscope. The experiments were repeated three times, and standard deviations were calculated using Microsoft Excel.

### Imaging of virus localization in fixed silkworm cells

BmN-SWU1 and BmN-SWU2 cells were seeded on coverslips (Fisher Scientific, Waltham, MA, USA) in 24-well plates (Corning, Corning, NY, USA) and cultured at 27°C for 1 day before infection. Cells were inoculated with virus for 1 h as described above [23] and washed three times with medium. This time point was designated as time 0 h p.i. At 6 h p.i., the cells were fixed with 4% paraformaldehyde in PBS for 10 min at room temperature and washed three times with PBS. Fixed cells were permeabilized in 0.1%Triton X-100 in PBS for 10 min and washed three times again with PBS. Cells were blocked with 10% normal goat serum in PBS for 1.5 h at 37°C, followed by incubation with a mouse monoclonal α-EGFP antibody (1∶200, Abcam, Cambridge, MA, USA) for 1 h at 37°C. After washing six times with PBS, cells were reacted with an AlexaFluor555-conjugated goat α-mouse IgG antibody for 1 h, stained with 0.1 µg/ml DAPI (Sigma, St. Louis, MO, USA), and finally washed by six times in PBS. All cells were visualized with an Olympus BX21 fluorescence microscope (Olympus, Tokyo, Japan).

### DNA replication assay

To analyze viral DNA replication, BmN-SWU1 or BmN-SWU2 cells were transfected with 1 µg of recombinant vA4^prm^-VP39-EGFP bacmid DNA. At selected time points (0, 12, 24, 48, 72 and 96 h p.t.), the cells were washed three times with PBS and collected. Total DNA was prepared with the Wizard Genomic DNA Purification Kit (Promega, Madison, WI, MA, USA) according to the manual's protocol. Prior to PCR, 1 µg of total DNA from each time point was digested with 1 µl *Dpn*I at 37°C for 4 h to eliminate input methylated bacmid DNA. The BmNPV *GP41* gene was used to detect viral DNA abundance. The silkworm *GAPDH* gene was used for normalization. Sequences of primers used were as follows: GP41 forward 5′-CGTAGTAGTAGTAATCGCCGC-3′ and reverse 5′ -AGTCGAGTCGCGTCGCT TT-3′); GAPDH forward 5′-CATTCCGCGTCCCTGTTGCTAAT-3′ and reverse 5′-GCTGCCTCCTTGACCTTTTGC-3′. Quantitative real-time PCR (Q-PCR) was performed using an ABI PRISM 7000 sequence detection system (Applied Biosystems, Carlsbad, CA, USA). Amplification was carried out in a 15 µl reaction mixture containing 20 ng of DNA sample, 0.5 mM of each primer and 1× SYBR Premix Ex Taq (TaKaRa, Dalian, China) in each well of a 96-well plate. The reaction conditions were 94°C for 4 min, followed by 40 cycles at 95°C for 10 s, 60°C for 20 s, 72°C for 45 s, and 72°C for 10 min. Each assay was repeated three times. The results were normalized to copies of the *GAPDH* gene DNA. Student's *t*-test was used to evaluate statistical significance (P<0.01).

### Reverse transcription quantitative PCR (RT-qPCR)

Total RNA from each sample was extracted at selected time points as previously described. Genomic DNA was removed using RQ1 RNase-Free DNase (Promega). Reverse transcription was carried out using MLV-RT (Promega). The silkworm housekeeping gene *sw22934* was used as an internal control. RT-qPCR was performed using the StepOne PlusTM Real-time System (Applied Biosystems). Ampfication was carried out in a 15 µl reaction mixture containing 1 µl of cDNA, 0.5 mM of each primer and 2×SYBR Select Master Mix (Life Technology, Carlsbad, CA, USA) in each well of a 96-well plate. The reaction procedure was 94°C 10 s, followed by 40 cycles at 95°C for 5 s and 60°C for 40 s. To confirm specific amplification, melting curve analysis was performed. Each expression assay was repeated three times. Student's *t*-test was used to evaluate statistical significance (P<0.01). Sequences for primers used were as follows: *ie-1* forward 5′ TACTTGGACGATTCACAAAG 3′ and reverse 5′ GTGCAAATGTTCGTGTTGTG 3′; *vp39* forward 5' ACTTTTCATGATGTCACTGC3' and reverse 5' AGTACTTGCAAATCGACACG 3'; *p10* forward 5' GACACGAATTTTAGACGCCATT 3' and reverse 5' CGATTCTTCCAGCCCGTTT 3'; *sw22934* forward 5' TTCGTACTGGCTCTTCTCGT 3' and reverse 5' CAAAGTTGATAGCAATTCCCT 3'.

### Western blotting

At 48 h post-transfection (p.t.), cells were washed twice with PBS and lysed with 100 µl protein lysis buffer (Beyotime, Shanghai, China) containing 1% PMSF protease inhibitors (Beyotime). After 30 min on ice, lysates were centrifuged at 12 000×g for 15 min and the supernatants were collected. The concentration of proteins in the supernatant was then measured using a BCA Protein Assay Kit (Beyotime). Approximately 25 µg of total proteins were resolved by 12% SDS-PAGE and analyzed by immunoblotting by using mouse monoclonal α-GP64 antibody (1∶2000, Abcam), mouse monoclonal α-EGFP antibody (1∶2000, Abcam) and mouse monoclonal α-tubulin antibody (1∶2000, Beyotime). The horseradish peroxidase-conjugated goat α-mouse IgG (1∶20000, Beyotime) was used as the secondary antibody. Protein bands were visualized using the Lumi-Light PLUS Western Blotting Kit (Roche) and a Chemiluminescence Imaging System (Clinx Science Instruments, Shanghai, China).

### Syncytium formation assay

To generate transient expression vectors, a fragment of the BmNPV *GP64* gene with flanking *Eco*RI and *Spe*I sites was amplified using BmNPV genomic DNA as a template and inserted into an *Eco*RI-*Xba*I digested pIZT/V5 plasmid (Invitrogen). The resulting GP64 expression vector containing the *EGFP* gene was named pIZT-GP64. The pIZT/V5 vector is a dual expression system (Invitrogen) which is used to express one gene of interest and a GFP-fused Zeocin antibiotic resistance gene. Both expression cassettes are under control of the constitutive OpIE2 promoter. After transfection into insect cells, pIZT-GP64 expressed both GP64 protein and GFP fluorescent protein. Thus, the green florescence was used to monitor GP64-expressing cells. Syncytium formation assays were performed by transfection with 1 µg of the pIZT-GP64 plasmid into BmN-SWU1 or BmN-SWU1 cells. At 48 h p.t., cells were washed twice with 1 ml TC-100 medium (pH 6.19) and treated for 10 min with acidified TC-100 medium (pH 4.50). The acidic medium was removed and replaced with 2 ml of normal TC-100 medium (pH 6.19) with 10% FBS. Syncytium was examined 12 h later by indirect immunofluorescence with primary antibodies α-GP64 and α-EGFP. Nuclei were stained with DAPI (blue).

## Results

### Identification of silkworm ovarian cell lines

**Figure 1:**
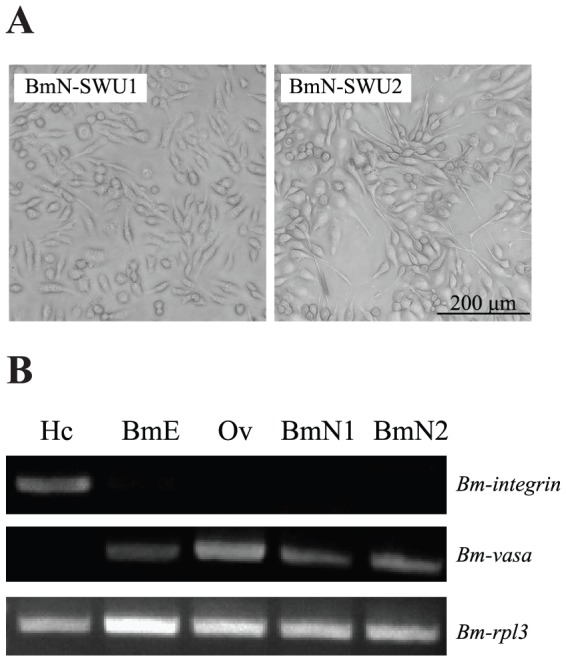
Characterization of BmN-SWU1 and BmN-SWU2 ovarian cell lines. (A) Observation of BmN-SWU1 and BmN-SWU2 cell lines established from larval ovaries of *B.mori* under a phase contrast microscope. (B) Semi-quantitative RT-PCR to detect gene expression patterns in tissues and subcultured cell lines. Hc, hemocytes; BmE, BmE-SWU1 (silkworm embryonic cell line); Ov, ovary; BmN1, BmN-SWU1; BmN2, BmN-SWU2.

We established the ovarian cell line BmN-SWU1 in our previous study [21]. Another novel cell line, BmN-SWU2, was obtained from the primary culture of the same tissue source. These two cell lines showed different morphological characteristics. The BmN-SWU1 cell line mainly appeared as elliptical and spindle-like cells (passage >500), while the BmN-SWU2 contained a large number of long needle-shaped cells in addition to round cells (Figure 1A). Population doubling times of BmN-SWU1 and BmN-SWU2 cells were approximately 60 h and 96 h, respectively. The expression patterns of *Bm-integrin* and *Bm-vasa* were analyzed to confirm the origin of these ovarian cell lines. The results showed that *Bm-integrin* was specifically expressed in hemocytes and at a low level in the embryo cell line BmE-SWU1. *Bm-vasa* mRNA was detected not only in *B. mori* ovarian tissue, BmE-SWU1 embryonic cells, but also in BmN-SWU1 and BmN-SWU2 ovarian cells. By contrast, *Bm-integrin* was not expressed in any of the cell lines or the ovarian tissue (Figure 1B). These results indicated that BmN-SWU1 and BmN-SWU2 were both derived from a silkworm ovarian tissue and not from contaminating hemocytes.

### Susceptibility of ovarian cell lines to BmNPV infection

**Figure 2:**
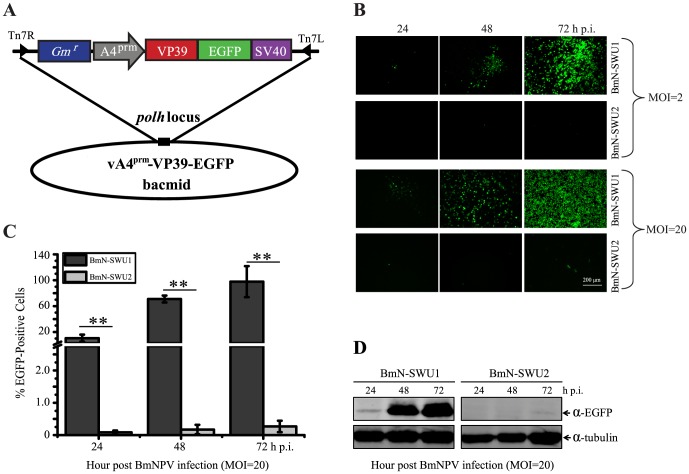
Comparison of susceptibilities of BmN-SWU1 and BmN-SWU2 ovarian cell lines to BmNPV infection. (A) Schematic diagram illustrating construction of donor plasmids and generation of recombinant BmNPV vA4^prm^-VP39-EGFP, which contains the *EGFP* fluorescence marker gene driven by the silkworm *actinA4* promoter. (B) Fluorescence microscopy showing the progression of infection in both cells lines infected with vA4^rpm^-VP39-EGFP (MOI = 2 or 20) at 24, 48 and 72 h p.i. (C) BmNPV infection efficiencies (EGFP positive, EGFP+) in BmN-SWU1 and BmN-SWU2 cells were determined by counting fluorescent cells in five different fields of view. Three independent experiments were carried out with three replicates each. Data are presented as means ± SD (n = 3). Statistically significant differences: **P<0.01. (D) Western blot analysis of silkworm ovarian cells lysed at indicated times after vA4^rpm^-VP39-EGFP infection (MOI = 20) using an α-EGFP antibody.

Here, to our surprise, very different responses were displayed between BmN-SWU1 and BmN-SWU2 cells when exposed to BmNPV. The BmN-SWU1 cell line showed typical cytopathogenic effects (CPE) of BmNPV infection, such as uniform rounding and distinctly enlarged nuclei. However, no obvious characteristics of infection were observed in BmN-SWU2 cells. The infection efficiencies of BmNPV in the two cell lines were quantified using the recombinant vA4^prm^-VP39-EGFP containing an EGFP reporter gene (Figure 2A). EGFP-positive cells were counted under a fluorescence microscope at various time points, and data were reported as a percentage of EGFP-expressing cells. The results showed massive replication of BmNPV in BmN-SWU1 cells, while few BmN-SWU2 cells exhibited EGFP expression (Figure 2B). As shown by quantitative analysis, EGFP expression in the BmN-SWU1 cell line inoculated with BmNPV (MOI = 20) was detected at 24 h p.i., when approximately 19.3% of the cells were positive. A significant increase in EGFP expression was observed at 48 h p.i., and the maximum level of approximately 97.8% was reached at 72 h p.i. By contrast, few BmNPV-inoculated BmN-SWU2 cells expressed EGFP at 24 h p.i., and the infection efficiency was only 0.08%. At 48 and 72 h p.i., the rates of EGFP-positive cells increased only slightly up to 0.17% and 0.26% in virus-exposed BmN-SWU2 cells, respectively (Figure 2B and 2C). Western blotting also revealed a minimal or undetectable level of EGFP expression in BmNPV-inoculated BmN-SWU2 cells compared with BmN-SWU1 cells. Thus, BmN-SWU2 was determined to be a BmNPV-nonpermissive cell line and a suitable cell model for silkworm anti-BmNPV research.

### Visualization of virus entry into BmN-SWU1 and BmN-SWU2 cells

**Figure 3:**
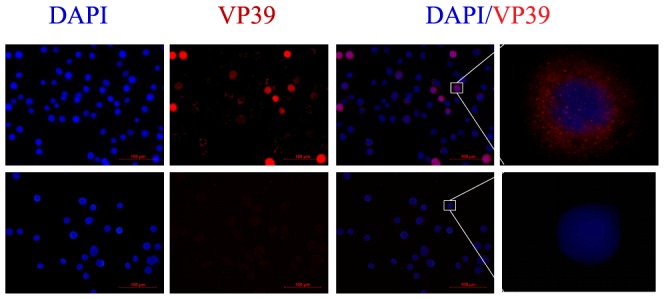
Detection of baculovirus entry into silkworm ovarian cells. Localizations of virions in infected BmN-SWU1 and BmN-SWU2 cells were detected by indirect immunofluorescence at 6 h p.i. VP39-EGFP was detected using a mouse α-EGFP monoclonal antibody and AlexaFluor555-conjugated goat α-mouse IgG antibody (red). Nuclei were stained with DAPI (blue). The merged image was generated using SPOT Advance software.

Whether BmNPV particles could be imported into the BmN-SWU2 cell line was examined with the objective of identifying the step in which the infection process is impaired in those cells. Recombinant vA4^prm^-VP39-EGFP BVs were adsorbed to BmN-SWU1 or BmN-SWU2 cells and incubated for 6 h at 27°C. After fixing the cells, virions were tracked by detecting VP39-EGFP fusion protein signals via indirect immunofluorescence could amplify the target viral signals. As expected, strong viral particles were detected in the cytoplasm and nucleus of BmNPV-infected BmN-SWU1 cells, and gathered at the periphery of the nuclear membrane. By contrast, no viral capsid signals were observed in BmNPV-inoculated BmN-SWU2 cells (Figure 3). These results indicate that the entry of BmNPV BVs was inhibited by yet unknown mechanisms after inoculation of BmN-SWU2 cells.

### Analysis of viral DNA replication and viral gene transcription

**Figure 4:**
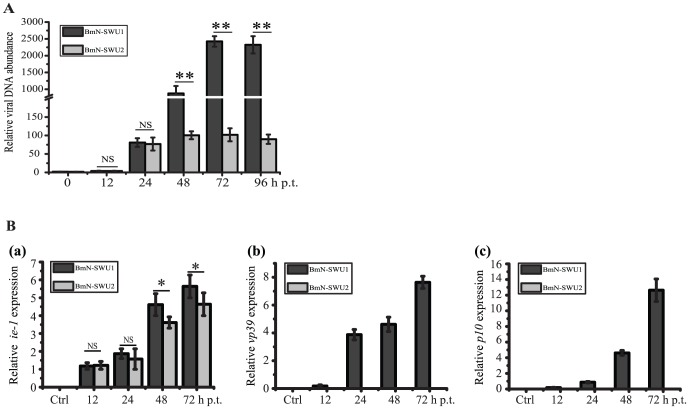
Viral DNA replication and gene transcription in cells transfected with vA4^prm^-VP39-EGFP bacmid DNA. (A) Total cellular DNA was extracted from BmN-SWU1 and BmN-SWU2 cells at indicated times after vA4^prm^-VP39-EGFP viral DNA transfection and analyzed by Q-PCR using GP41 DNA primers as described in methods. Relative BmNPV genomic copy numbers were calculated using *B. mori* GADPH DNA as an internal control. Results are mean values of samples from each time point tested in triplicate. NS, not significant. Statistically significant differences: *P<0.05. (B) RT-qPCR analysis of viral gene transcription levels after vA4^prm^-VP39-EGFP viral DNA transfection. a, Variation of early gene *ie-1* transcription levels in transfected ovarian cell lines over time. b, The variation of late gene *vp39* transcription level in transfected ovarian cell lines over time. c, Variation of very late gene *p10* transcription levels in transfected ovarian cell lines over time. Values are expressed as the mean ± SEM. Similar results were obtained in three independent experiments. Ctrl, negative control (without reverse transcription). NS, not significant. Statistically differences: *P<0.05.

Upon release of the nucleocapsid into the host nucleus, the baculovirus genomic DNA begins replication, and inhibition of viral DNA synthesis can reduce yields of BVs by more than 1,000-fold [24]. Although few BmNPV particles could infect BmN-SWU2 cells in this study, learning whether the viral DNA could replicate normally upon successful entry was a point of interest. Hence, Q-PCR was used to monitor viral DNA replication of BmNPV in BmN-SWU1 and BmN-SWU2 cells after transfection with viral bacmid DNA (Figure 4A). At 0 h p.t., very little viral DNA was detected in either transfected cell line, indicating equal amounts of input DNA. At 24 h p.t., the onset of viral DNA replication was detected in both the susceptible BmN-SWU1 cells and the resistant BmN-SWU2 cells, with a relative DNA abundance of 80.82 and 76.37 fold respectively. From 24 to 96 h p.t., a substantial and steady increase of viral DNA replication was found in BmN-SWU1 cells. By contrast, no significant increase of viral DNA was observed in the transfected BmN-SWU2 cells. This result indicated that BmNPV bacmid DNA could initiate replication normally in the BmN-SWU2 cells after transfection. In other words, if BmNPV was imported into the cell membrane of BmN-SWU2 cells, initiation of viral DNA replication would occur normally as it would in BmNPV-susceptible cells. Based on the temporal expression of the viral genes, the infection cycle can be subdivided into three major phases: early, late, and very late. Some of the early genes encode products necessary for DNA replication and late gene expression. To determine BmNPV gene expression in BmN-SWU2 cells, we investigated the transcription of early gene *ie-1*, late gene *vp39* and very late gene *polh* in BmN-SWU1 and BmN-SWU2 cells after transfection with viral bacmid DNA. The results showed that the transcription of the *ie-1* early gene did not differ significantly in the two cell lines at 24 h p.t. but the its transcription level in BmN-SWU1 cells were higher than that in BmN-SWU2 cells at 48 h p.t. Moreover, late and very late viral genes were expressed in the BmN-SWU1 cell lines, but not in the resistant BmN-SWU2 cell line (Figure 4B). All these results indicated that BmN-SWU2 cells could support transcription of viral early genes and viral DNA replication, but not transcription of late and very late viral genes.

### Membrane fusion analysis

**Figure 5:**
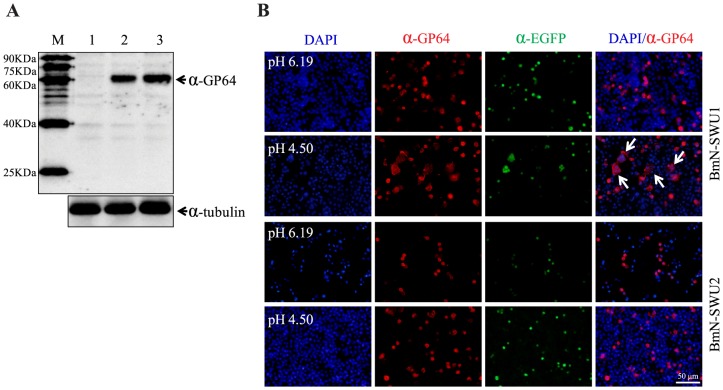
Detection of GP64 expression and fusogenicity. (A) Western blot analysis of GP64 expression in cells transfected with 1 µg of the pIZT-GP64 plasmid or a control pIZT/V5 plasmid. At 48 h p.t., proteins were separated by SDS-PAGE. The blots were probed with α-GP64 antibody or α-tubulin antibody. M: protein marker; lane 1, BmN-SWU1 cells transfected with 1 µg pIZT/V5 plasmid; lane2, BmN-SWU1 cells transfected with 1 µg pIZT-GP64 plasmid; lane3, BmN-SWU2 cells transfected with 1 µg pIZT-GP64 plasmid. (B) Syncytium formation assay of BmN-SWU1 or BmN-SWU2 cells transfected with 1 µg pIZ-GP64 plasmid. At 48 h p.t., cells were incubated in TC-100 medium at a normal (6.19) or low (4.8) pH level for 10 min. Syncytium formation was examined 12 h later by detecting GP64 and EGFP using indirect immunofluorescence microscopy. Nuclei were stained with DAPI (blue).

Because the viral-cell membrane interaction is a key step prior to the import of BmNPV into the cytoplasm of the host cell, it is possible that a defect in this process contributes to a block in the transport of BVs into BmN-SWU2 cells. GP64 is an important envelope protein that interacts with the host receptor and triggers viral envelope fusion with the host cellular plasma membrane in an acidic environment. In the current study, we conducted syncytium formation assays to investigate whether GP64 could mediate virus-host interaction in BmN-SWU2 cells. As described in Materials and methods, the GP64 expression plasmid was constructed and confirmed by Western blot analysis. Using α-GP64 antibody, a band corresponding to the predicted molecular size of BmNPV GP64 (60.6 KDa) was detected in BmN-SWU1 or BmN-SWU2 cells transfected with pIZT-GP64 (Figure 5A, lanes 2 and 3), but not in BmN-SWU1 cells transfected with the pIZT/V5 control plasmid. Subsequently, cells transfected with the pIZT-GP64 plasmid were exposed to low pH and observed under a light microscope. Multinuclear cells were observed in BmN-SWU1 cells transfected with pIZT-GP64, demonstrating that GP64 could cause cell-cell membrane fusion in a low-pH environment in this cell line (Figure 5B). However, cell-to-cell fusion was not observed in low-pH treated BmN-SWU2 cells transfected with the pIZT-GP64 plasmid, demonstrating that GP64 could not induce intercellular membrane fusion of these cells (Figure 5B). These results suggest that the abortive replication of BmNPV in BmN-SWU2 cells is mainly due to a disruption of the direct interaction of viral GP64 with the host membrane.

## Discussion

The ovary of *B. mori* is known to be a susceptible tissue to BmNPV infection [25]. However, two *B. mori* cell lines in this study, BmN-SWU1 and BmN-SWU2, which were isolated from the same ovarian tissue source, demonstrated surprisingly very different responses when inoculated with recombinant BmNPV. BmN-SWU1 cells apparently were susceptible to BmNPV infection. However, BmNPV-inoculated BmN-SWU2 cells showed no obvious characteristics of infection (Figure 2). To our knowledge, the current study is the first to report an ovary-derived cell line which is resistant to BmNPV infection. BmNPV has previously shown distinct tissue or cell tropism (i.e., BmNPV replicating poorly in the anterior silkglands, midgut, Malpighian tubule, salivary glands and plasmatocytes) [4], [26], [27]. In this study, expression patterns of several genes were analyzed to identify the ovarian cell lines. Because *Bm-integrin* is expressed in hemocytes and not in other tissues, it is considered to be one of the most reliable molecular markers of hemocytes. The current results showed that *Bm-integrin* was not expressed in either BmN-SWU1 or BmN-SWU2 cells, indicating that these two cell lines were not derived from hemocytes. VASA is considered to be one of the key regulatory protein factors of insect germ cells and a marker of germ cells [28]. The fact that *Bm-vasa* was detected in the BmE-SWU1 embryonic cells, as well as in the BmN-SWU1 and BmN-SWU2 cell lines, but not in hemocytes, confirmed that the latter two cell lines were derived from ovarian cells and not from contaminating hemocytes.

To determine the possible steps at which BmNPV replication was blocked in BmN-SWU2 cells, we first analyzed the subcellular localization of virus particles in both ovarian cell lines. Our results revealed a major defect in the steps during the entry of BmNPV (Figure 3). Baculovirus entry into host cells is typically initiated by an interaction between the viral envelope glycoprotein GP64 and a host cell receptor [6], [9], [29]. GP64 then mediates the low-pH-triggered membrane fusion activity necessary for release of the nucleocapsids into the cytosol during entry by endocytosis [29]. Hence, analysis of the syncytium formation induced by expressing GP64 from a plasmid is an effective approach to investigating virus-host interaction and uncovering the nature of cellular factors required for the fusion. In the current study, we conducted the syncytium formation assay by transfecting cells with the pIZT-GP64 plasmid. After expression, GP64 molecules were transported to the surface of the plasma membrane where they could cause the host cell plasma membrane to fuse with neighboring cells and form multinuclear syncytia in the low-pH condition. Cell-to-cell fusion was observed in the BmNPV permissive BmN-SWU1 cell line, but not in the nonpermissive BmN-SWU2 cells (Figure 5B). This result suggests that the block in entry of BmNPV into BmN-SWU2 cells is mainly due to a defective interaction with the host plasma membrane. Because the defect in interaction could be in the binding or membrane fusion process, further research is needed to determine the exact step at which BmNPV entry is blocked in these cells and investigate cell surface molecules which are necessary for BmNPV-host interaction.

To investigate other steps affecting BmNPV pathogenicity to BmN-SWU2 cells, we subsequently analyzed viral DNA replication and viral gene transcription in BmN-SWU1 and BmN-SWU2 cells by a transfection method. The results showed a defect in the transcription of viral late genes in BmN-SWU2 cells, but normal transcription of early genes (Figure 4B). Baculovirus early genes are transcribed by the host RNA polymerase II and late and very late genes are transcribed by a virus-specific RNA polymerase which is composed of four subunits, LEF-4, LEF-8, LEF-9, and p47 [30]. Early gene products are necessary for viral DNA replication, while transcription of late and very late genes depend on DNA replication and viral transactivators. Baculovirus DNA replication can be described as a process of two stages, initiation and amplification. Q-PCR results demonstrated that BmNPV genome DNA could initiate replication, but it did not amplify significantly in transfected BmN-SWU2 cells (Figure 4A). A previous study revealed that, without amplification, initiation of viral DNA replication is not sufficient to support transcription of baculovirus late and very late genes [31]. All results above suggest that a host restriction factor may inhibit baculovirus DNA replication amplification, followed by blocking late genes transcription in BmN-SWU2 cells. In the future studies, we will perform a genome-wide high-content RNAi screen in BmN-SWU1 cells as a systematic approach to identify such cellular factors that may potentially limit BmNPV replication.

In conclusion, we characterized two insect cell lines (BmN-SWU1 and BmN-SWU2) from *B. mori* ovaries, which showed differential responses to BmNPV infection. Our results indicated that abortive replication of BmNPV in BmN-SWU2 ovarian cells was due to defects in BmNPV-host interaction and viral entry process. In addition, amplification after initiation of viral DNA replication and late gene transcription was also blocked in BmN-SWU2 cells. Thus, these two ovarian cell lines are ideal tools for identifying the BmNPV receptor protein in the host and investigating insect antiviral mechanisms. In addition, they may provide an excellent platform for studying resistance mechanisms of insects against baculovirus-based insecticides.

## References

[1] Tomita M, Munetsuna H, Sato T, Adachi T, Hino R, et al (2003) Transgenic silkworms produce recombinant human type III procollagen in cocoons. Nature Biotechnology 21: 52–56. 10.1038/nbt771 12483223

[2] Xia Q, Zhou Z, Lu C, Cheng D, Dai F, et al (2004) A draft sequence for the genome of the domesticated silkworm (*Bombyx mori*). Science 306: 1937–1940. 15591204 10.1126/science.1102210

[3] Mita K, Kasahara M, Sasaki S, Nagayasu Y, Yamada T, et al (2004) The genome sequence of silkworm, *Bombyx mori* . DNA Research 11: 27–35. 15141943 10.1093/dnares/11.1.27

[4] Rahman MM, Gopinathan KP (2004) Systemic and in vitro infection process of *Bombyx mori* nucleopolyhedrovirus. Virus Research 101: 109–118. 15041178 10.1016/j.virusres.2003.12.027

[5] Keddie BA, Aponte GW, Volkman LE (1989) The pathway of infection of *Autographa californica* nuclear polyhedrosis virus in an insect host. Science 243: 1728–1730. 2648574 10.1126/science.2648574

[6] Hefferon KL, Oomens AG, Monsma SA, Finnerty CM, Blissard GW (1999) Host cell receptor binding by baculovirus GP64 and kinetics of virion entry. Virology 258: 455–468. 10366584 10.1006/viro.1999.9758

[7] Monsma SA, Oomens AG, Blissard GW (1996) The GP64 envelope fusion protein is an essential baculovirus protein required for cell-to-cell transmission of infection. Journal of Virology 70: 4607–4616. 8676487 10.1128/jvi.70.7.4607-4616.1996 PMC190397

[8] Markovic I, Pulyaeva H, Sokoloff A, Chernomordik LV (1998) Membrane fusion mediated by baculovirus gp64 involves assembly of stable gp64 trimers into multiprotein aggregates. Journal of Cell Biology 143: 1155–1166. 9832546 10.1083/jcb.143.5.1155 PMC2133075

[9] Zhou J, Blissard GW (2008) Identification of a GP64 subdomain involved in receptor binding by budded virions of the baculovirus *Autographica californica* multicapsid nucleopolyhedrovirus. Journal of Virology 82: 4449–4460. 18287233 10.1128/JVI.02490-07 PMC2293031

[10] Li Z, Blissard GW (2009) The *Autographa californica* multicapsid nucleopolyhedrovirus GP64 protein: analysis of transmembrane domain length and sequence requirements. Journal of Virology 83: 4447–4461. 19244324 10.1128/JVI.02252-08 PMC2668483

[11] Yang Y, Tang L, Tong L, Liu H (2009) Silkworms culture as a source of protein for humans in space. Advances in Space Research 43: 1236–1242.

[12] Kadlec J, Loureiro S, Abrescia NG, Stuart DI, Jones IM (2008) The postfusion structure of baculovirus gp64 supports a unified view of viral fusion machines. Nature Structural & Molecular Biology 15: 1024–1030. 10.1038/nsmb.1484 18776902

[13] Luz-Madrigal A, Asanov A, Camacho-Zarco AR, Sampieri A, Vaca L (2013) A cholesterol recognition amino acid consensus domain in GP64 fusion protein facilitates anchoring of baculovirus to mammalian cells. Journal of Virology 87: 11894–11907. 23986592 10.1128/JVI.01356-13 PMC3807332

[14] Goley ED, Ohkawa T, Mancuso J, Woodruff JB, D'Alessio JA, et al (2006) Dynamic nuclear actin assembly by Arp2/3 complex and a baculovirus WASP-like protein. Science 314: 464–467. 17053146 10.1126/science.1133348

[15] Mueller J, Pfanzelter J, Winkler C, Narita A, Le Clainche C, et al (2014) Electron tomography and simulation of baculovirus actin comet tails support a tethered filament model of pathogen propulsion. PLoS Biology 12: e1001765. 24453943 10.1371/journal.pbio.1001765 PMC3891563

[16] Ohkawa T, Volkman LE, Welch MD (2010) Actin-based motility drives baculovirus transit to the nucleus and cell surface. Journal of Cell Biology 190: 187–195. 20660627 10.1083/jcb.201001162 PMC2930276

[17] Tjia ST, Carstens EB, Doerfler W (1979) Infection of *Spodoptera frugiperda* cells with A*utographa californica* nuclear polyhedrosis virus II. The viral DNA and the kinetics of its replication. Virology 99: 399–409. 16945843 10.1016/0042-6822(79)90018-7

[18] Clarke TE, Clem RJ (2003) In vivo induction of apoptosis correlating with reduced infectivity during baculovirus infection. Journal of Virology 77: 2227–2232. 12525657 10.1128/JVI.77.3.2227-2232.2003 PMC140947

[19] Katsuma S, Tsuchida A, Matsuda-Imai N, Kang W, Shimada T (2011) Role of the ubiquitin-proteasome system in *Bombyx mori* nucleopolyhedrovirus infection. Journal of General Virology 92: 699–705. 21084493 10.1099/vir.0.027573-0

[20] Xue J, Qiao N, Zhang W, Cheng RL, Zhang XQ, et al (2012) Dynamic interactions between *Bombyx mori* nucleopolyhedrovirus and its host cells revealed by transcriptome analysis. Journal of Virology 86: 7345–7359. 22532689 10.1128/JVI.07217-12 PMC3416345

[21] Pan MH, Cai XJ, Liu M, Lv J, Tang H, et al (2010) Establishment and characterization of an ovarian cell line of the silkworm, *Bombyx mori* . Tissue Cell 42: 42–46. 19665160 10.1016/j.tice.2009.07.002

[22] Wang F, Xu H, Yuan L, Ma S, Wang Y, et al.. (2013) An optimized sericin-1 expression system for mass-producing recombinant proteins in the middle silk glands of transgenic silkworms. Transgenic Research. 10.1007/s11248-013-9695-6 23435751

[23] Katou Y, Ikeda M, Kobayashi M (2006) Abortive replication of *Bombyx mori* nucleopolyhedrovirus in Sf9 and High Five cells: defective nuclear transport of the virions. Virology 347: 455–465. 16412489 10.1016/j.virol.2005.11.043

[24] Kool M, Ahrens CH, Goldbach RW, Rohrmann GF, Vlak JM (1994) Identification of genes involved in DNA replication of the *Autographa californica* baculovirus. Proceedings of the National Academy of Sciences of the United States of America 91: 11212–11216. 7972036 10.1073/pnas.91.23.11212 PMC45197

[25] Khurad AM, Mahulikar A, Rathod MK, Rai MM, Kanginakudru S, et al (2004) Vertical transmission of nucleopolyhedrovirus in the silkworm, *Bombyx mori* L. Journal of Invertebrate Pathology 87: 8–15. 15491594 10.1016/j.jip.2004.05.008

[26] Katsuma S, Kobayashi J, Koyano Y, Matsuda-Imai N, Kang W, et al (2012) Baculovirus-encoded protein BV/ODV-E26 determines tissue tropism and virulence in lepidopteran insects. Journal of Virology 86: 2545–2555. 22190721 10.1128/JVI.06308-11 PMC3302242

[27] Hori T, Kiuchi T, Shimada T, Nagata M, Katsuma S (2013) Silkworm plasmatocytes are more resistant than other hemocyte morphotypes to *Bombyx mori* nucleopolyhedrovirus infection. Journal of Invertebrate Pathology 112: 102–104. 23026703 10.1016/j.jip.2012.09.004

[28] Raz E (2000) The function and regulation of vasa-like genes in germ-cell development. Genome Biology 1(3) : reviews 1017.1–1017.6. 10.1186/gb-2000-1-3-reviews1017 PMC138859 11178242

[29] Blissard GW, Wenz JR (1992) Baculovirus gp64 envelope glycoprotein is sufficient to mediate pH-dependent membrane fusion. Journal of Virology 66: 6829–6835. 1404622 10.1128/jvi.66.11.6829-6835.1992 PMC240187

[30] Acharya A, Gopinathan KP (2002) Characterization of late gene expression factors lef-9 and lef-8 from *Bombyx mori* nucleopolyhedrovirus. Journal of General Virology 83: 2015–2023. 12124466 10.1099/0022-1317-83-8-2015

[31] Wu CP, Huang YJ, Wang JY, Wu YL, Lo HR, et al (2010) *Autographa californica* multiple nucleopolyhedrovirus LEF-2 is a capsid protein required for amplification but not initiation of viral DNA replication. Journal of Virology 84: 5015–5024. 20219928 10.1128/JVI.02423-09 PMC2863805

